# Cloned Pig Fetuses Have a High Placental Lysophosphatidylcholine Level That Inhibits Trophoblast Cell Activity

**DOI:** 10.3390/jdb13040041

**Published:** 2025-11-12

**Authors:** Junkun Lai, Xiaoyu Gao, Guke Zhang, Xiao Wu, Yiqian Zhang, Shunbo Wang, Zhenfang Wu, Zicong Li, Zheng Xu

**Affiliations:** 1 Guangdong Laboratory for Lingnan Modern Agriculture, South China Agricultural University, Guangzhou 510642, China; junkun_lai1996@163.com (J.L.); 15688771757@163.com (X.G.); gukezhang@stu.scau.edu.cn (G.Z.); wuxiao5901@163.com (X.W.); zyqhah-@stu.scau.edu.cn (Y.Z.); 03028301@stu.scau.edu.cn (S.W.); wzf@scau.edu.cn (Z.W.); 2 National Engineering Research Center for Breeding Swine Industry, South China Agricultural University, Guangzhou 510642, China; 3 State Key Laboratory of Swine and Poultry Breeding Industry, South China Agricultural University, Guangzhou 510642, China; 4 National and Local Joint Engineering Research Center for Livestock and Poultry Breeding Industry, South China Agricultural University, Guangzhou 510642, China; 5 Guangdong Provincial Key Lab of Agro-Animal Genomics and Molecular Breeding, South China Agricultural University, Guangzhou 510642, China; 6 Gene Bank of Guangdong Local Livestock and Poultry, South China Agricultural University, Guangzhou 510642, China; 7 College of Animal Science, South China Agricultural University, Guangzhou 510642, China

**Keywords:** cloned, placenta, trophoblast, LPC, transcriptome, lipidomics

## Abstract

Somatic cell nuclear transfer (SCNT) or cloning technology is widely used in agriculture and biomedicine. However, the application of this technology is limited by the low developmental competence of cloned embryos or fetuses, which frequently exhibit abnormal development of trophoblast cells or placentas. The purpose of this study was to investigate the possible causes of the erroneous placental development of SCNT-derived pig fetuses. The placental transcriptomic and lipidomic profiles were compared between 30-day-old SCNT- and artificial insemination (AI)-produced pig fetuses. Differentially expressed lipid metabolites between two groups of placentas were selected to test their effects on porcine trophoblast cell activity. The results showed that SCNT placentas exhibit impaired lipid metabolism and function. The level of a metabolite, lysophosphatidylcholine (LPC), in the glycerophospholipid metabolism pathway was substantially increased in SCNT placentas, compared with AI placentas. The elevation in LPC content may lead to impaired placental development in cloned pig fetuses, as LPC inhibited the proliferation and migration of porcine trophoblast cells. This study discovers a main cause of erroneous development of cloned pig fetuses, which will be beneficial for understanding the regulation of SCNT embryo development, as well as developing new methods to improve the efficiency of pig cloning.

## 1. Introduction

The somatic cell nuclear transfer (SCNT), which is also called cloning technology, is widely applied in agriculture and biomedicine [1,2,3,4,5]. However, the SCNT-generated embryos have a very low full-term developmental rate at about 1–5% [6,7,8], while the SCNT-produced animals have a high neonatal death rate at 48–74.5% [9,10,11]. The extremely low developmental competence of cloned embryos and animals hinders the application of the SCNT technology.

The trophoblast stem cell-derived placenta is an essential organ for the developing fetus. It is responsible for the exchange of nutrients, oxygen and metabolic waste between the mother and the fetus [12,13,14]. Placental dysfunction can lead to a variety of pregnancy complications, such as abortion, intrauterine growth retardation and stillbirth [15,16,17]. Abnormal placental histomorphology, such as reduction in placental leaf area and blood vessels, and dysplasia of placental villi, was frequently observed in cloned fetuses [18,19,20,21]. Erroneous gene expression was also found in the placenta of cloned fetuses [22,23,24,25,26]. When the defective trophoblast stem cells of mouse SCNT embryos were replaced by fertilization-derived fully functional trophoblasts, the birth rate of SCNT embryos was significantly increased by sixfold [27]. These studies indicated that functional defect of trophoblasts or placenta is one of the main reasons causing the low developmental efficiency of cloned embryos. However, the molecular mechanisms underlining placental dysplasia in SCNT fetuses remain unclear.

This study aimed to explore the factors leading to the abnormal placental development of porcine SCNT embryos, by comparing the placental transcriptomic and lipidomic profiles of 30-day-old SCNT and artificial insemination (AI)-derived pig fetuses. We found that the abnormally elevated placental lysophosphatidylcholine (LPC) level is related to the impaired development of placentas in pig SCNT fetuses.

## 2. Materials and Methods

### 2.1. Production of SCNT and AI Fetuses

The animal experimental protocols used in this study were approved by the Institutional Animal Care and Use Committee of South China Agricultural University (approval number: 2020f005). Five Duroc sows showing natural signs of estrus were artificially inseminated three times, with an interval of 12 h, to produce AI fetuses. The interval between each insemination was 8 to 12 h, and each dose of semen contained 3 billion spermatozoa in 80 mL of semen.

SCNT fetuses were produced as previously reported [9]. The ear Donor fibroblasts used for SCNT were isolated from the ear portion of the same boar that provides semen to produce AI fetuses. In vitro matured oocytes were placed in TCM199 culture medium containing 7.5 ug/mL cytochalasin B. An enucleation pipette was inserted into the oocyte zona pellucida and the first polar body was aspirated. Donor fibroblasts were digested with 0.25% trypsin and placed in oocyte manipulation drops, and individual donor cells were aspirated with a syringe needle and microinjected into the periplasmic space of the enucleated oocytes. Then, they were electrically fused with two consecutive DC pulses of 1.2 kV/cm for 30 s in a medium containing 250 mM mannitol, 0.1 mM CaCl_2_-2H_2_O, 0.1 mM MgCl_2_-6H_2_O, 0.5 mM HEPES, and 0.01% PVA. After the activation treatment, the reconstructed embryos were cultured for 4 h in PZM-3 culture medium containing cytochalasin B, after which they were transferred into fresh PZM-3 solution for continued incubation for 20 h. SCNT embryos were transferred into the bilateral oviducts of five Duroc sows in simultaneous estrus with 220–240 embryos per sow. Ultrasound was performed 1 month after embryo transfer to monitor pregnancy in recipient sows.

The AI and SCNT procedures were carried out in August 2021. The Duroc sows used for AI and the Duroc sows used as SCNT embryo recipients have a similar genetic background and physiological conditions. SCNT recipient sows and AI sows were raised under the same conditions.

### 2.2. Placenta Sample Collection and Fetus Body Length Measurement

Three pregnant sows in the SCNT group and three pregnant sows in the AI group underwent hysterectomy after anesthesia induction on the 30th day of pregnancy, and the uterus was quickly separated from the reproductive tract, transported to the laboratory in a freezer, and then cut off. Only 3 fetuses were randomly selected from each litter, which means that a total of 9 fetuses were collected from each group. The body length of each fetus was measured after cutting the umbilical cord. Immediately after the fetuses were removed, placental tissue samples were collected from the center of a single placenta and washed with phosphate-buffered saline before storage. All samples were immediately frozen in liquid nitrogen and stored at −80 °C until use.

### 2.3. RNA Sequencing

Total RNA of the samples was extracted, and cDNA was generated and amplified based on the smartseq2 method. Library quality was assessed using an AgilentBioanalyzer2100 system. Clustered library preparations were sequenced on the illuminaNova platform and 150 bp double-ended reads were generated. Clean reads were mapped to the Susscrofa11.1 reference genome by HISAT2 (v2.0.5). Differentially expressed gene (DEG) identification, Gene Ontology (GO) and Kyoto Encyclopedia of Genes and Genomes (KEGG) analysis were performed on a website in June 2022 (https://www.omicshare.com/).

### 2.4. Lipidomics Analysis

Aliquots of 200 mg of placental tissue samples were weighed and ground by liquid nitrogen. The samples had 50% methanol of 120 µL added to them and mixed thoroughly, followed by standing at room temperature for 10 min to extract the lipids from the samples. The extract was placed at −20 °C overnight to precipitate the proteins in the sample. The samples were centrifuged at 4000× *g* for 20 min. The supernatant was added to a 96-well plate and diluted with diluent (isopropanol: acetonitrile: water = 2:1:1, *v*/*v*). An aliquot of 10 µL of dilution was removed from each sample and was mixed into a QC sample. Six quality control (QC) samples were analyzed by ultra-high-performance liquid chromatography–mass spectrometry (LC-MS).

The raw files were converted to mzXML format using Proteowizard’s MSConvert 3.0 software in June 2022. The mzXML file is imported into the XCMS software for peak extraction. Metabolites were identified by metaX 3.0 software (primary mass spectrometry information for database matching and identification, secondary mass spectrometry information for matching and identification with in-house standards database), and identified candidate substances were metabolite-annotated utilizing HMDB and KEGG databases, respectively. The q-value was obtained by *t*-test statistical analysis, multiple test analysis of test results, and using BH correction. The importance VIP value of each metabolite variable was obtained by PCA and PLS-DA discriminant analysis. Significantly different metabolites were obtained by taking ratio > 2, VIP > 1 and *p* < 0.05 as screening conditions. The total ion chromatogram (TIC) and principal component analysis (PCA) score diagram of the quality control samples show that the chromatographic separation system has high accuracy, consistency and reproducibility (see Supplementary Figure S1).

### 2.5. qPCR

Placental tissue RNA was extracted using Total RNA KitI (R6934-02, Omega, GA, USA), and the RNA concentration was determined spectrophotometrically. Subsequently, RNA was reverse transcribed into cDNA using the reverse transcription kit PrimeScript RT Reagent Kit (RR037A, Takara, Tokyo, Japan). Expression levels of genes were quantified using the fluorescent quantitative PCR kit PowerUpTM SYBR Green Master Mix (A25742, Thermo Fisher, Boston, MA, USA). QPCR reactions were performed on a QuantStudio 7 Flex system (Thermo Fisher Scientific, Waltham, MA, USA). Gene expression levels were calculated according to the 2^−ΔΔCt^ formula using GAPDH as internal controls. Primer information has been provided (see Supplementary Table S1).

### 2.6. Cell Culture

PTr2 cells were isolated from porcine filamentous embryos of day 12 of gestation. PTr2 cells were cultured in DMEM/F12 (12634010, Gibco, Carlsbad, CA, USA) basal medium containing 10% fetal bovine serum (C0235, Gibco), 0.5% insulin (40112ES, YEASEN, Shanghai, China) and 1% penicillin–streptomycin (15140122, Gibco) at 37 °C with 5% CO_2_ cells to about 80–90% confluence. The cells were digested with 0.25% trypsin (25200056, Gibco) for 1 min at 37 °C, then terminated and centrifuged. The cells were resuspended in complete medium, counted with a cell counter, mixed and seeded into new cell culture plates.

### 2.7. Cell Counting Kit-8 (CCK-8) Assay

Cell suspensions were obtained as described in Section 2.6 and seeded 100 µL/well into 96-well plates. The number of seeded cells was adjusted to 1 × 10^5^ cells/mL, ensuring that the cells could grow to roughly 80–90% confluence in 48 h of culture. After incubation at 37 °C, 5% CO_2_ for 8 h, the culture medium was replaced with medium containing 2% low serum containing different concentrations of LPC (9008-30-4, Sigma). After 24 h, 36 h and 48 h of incubation, respectively, the 96-well plates were removed and CCK8 solution (40203ES60, YEASEN) was added, and then incubated in a cell culture incubator at 37 °C for 3 h, and the optical density (OD) of each well was detected by a multifunctional enzyme labeling instrument at a wavelength of 450 nm.

### 2.8. EdU Assay

Cell proliferation at different time points was detected using BeyoClickTMEDU-555 Cell Proliferation Detection Kit (C0071L, Beyotime, Shanghai, China). Cell suspensions were obtained and seeded into 96-well plates as described in Section 2.7. The number of seeded cells was adjusted to 1 × 10^5^ cells/mL ensure that the cells could grow to roughly 80–90% confluence at 48 h of culture. After incubation for 8 h at 37 °C, 5% CO_2_, the culture medium was replaced with medium containing 2% low serum containing different concentrations of LPC (9008-30-4, Sigma, St. Louis, MI, USA). After 24 h, 36 h and 48 h of incubation, respectively, the 96-well plate was taken out, and the prepared 2 × EDU working solution was added to the 96-well plate and incubated in a 37 °C, 5% CO_2_ cell incubator for 2 h, followed by removing the culture solution, and the cells were washed twice with DPBS for 5 min each time. The cells were fixed with 4% paraformaldehyde at room temperature for 30 min, and the fixation solution was discarded and washed with DPBS 3 times. Cells were permeabilized by adding permeabilizing solution for 10 min and washed with DPBS 3 times. Subsequently, the cells were incubated in a 96-well plate with Click reaction solution, 70 µL/well, at room temperature and protected from light for 35 min. Reaction solution was discarded and washed 3 times with DPBS. Nuclei staining was performed by adding 100 µL of Hoechst33342 per well and incubating for 30 min away from light. Subsequently, the staining solution was discarded and the cells were washed 3 times with DPBS. Fluorescence detection was performed under a fluorescence microscope.

### 2.9. Cell Migration Assay

Cell suspensions of one mL were seeded into 6-well plates as described in Section 2.7, and the number of seeded cells was adjusted to 6 × 10^5^ cells/mL to ensure that the cells could grow to roughly 80–90% confluence after 8 h of culture. After 8 h of incubation at 37 °C, 5% CO_2_, the cell culture solution was scratched vertically on the 6-well plate with a large pipette tip, and then the cell culture solution was changed to a serum-free culture solution with different concentrations of LPC (9008-30-4, Sigma, SL, USA), and put into the 37 °C, 5% CO_2_ constant temperature incubator for incubation. Cell migration and scratch healing were visualized at 0 h, 12 h and 24 h of culture, respectively, and 3 fields of view were randomly selected, and the healing area was calculated by ImageJ software (version 1.52) to calculate the cell migration rate [28].

### 2.10. Statistical Analysis

The data were analyzed and plotted using the GraphPad Prism 8.0. Student’s *t*-test was used for comparisons between two groups.

## 3. Results

### 3.1. Comparison of Body Length Between AI and SCNT Fetuses

**Figure 1:**
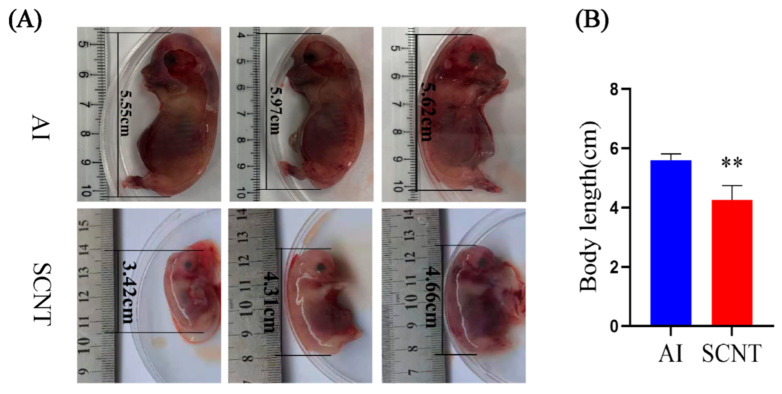
Comparison of body length between AI and SCNT fetuses. (**A**) Representative photos of 30-day-old fetuses from AI group and SCNT group. (**B**) Statistic analysis of body length of two groups. AI group: *n* = 9, SCNT group: *n* = 9. (** *p* < 0.01).

When we collected pig fetuses to isolate their placentas for analysis, we observed that the SCNT fetuses were smaller than the AI counterparts (Figure 1A). To confirm this, we measured and compared the body length of two groups of fetuses. The results indicated that the body length of the SCNT group (4.26 ± 0.45 cm) was significantly shorter than that of the AI group (5.58 ± 0.21 cm) (Figure 1B). In addition, the body color seems different between the two groups, as the body color of SCNT fetuses tended to be whiter than that of AI fetuses (Figure 1A). These data suggest that SCNT fetuses showed an abnormal development at the age of 30 days, compared with AI fetuses.

### 3.2. Transcriptome Analysis of AI and SCNT Fetal Placentas

**Figure 2:**
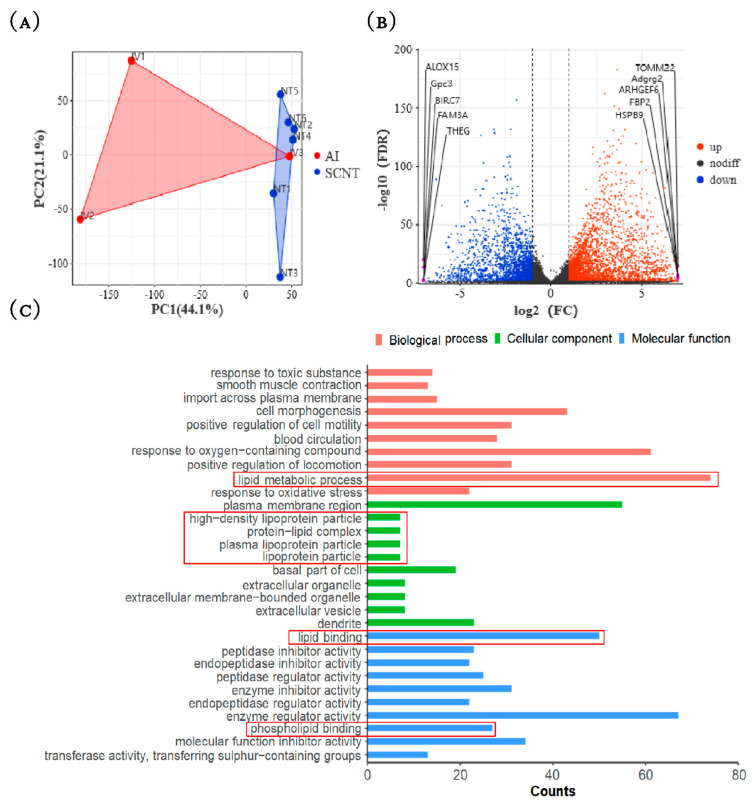
Transcriptome analysis of AI and SCNT fetal placentas. (**A**) PCA score scatter plot of two groups of samples. IV1 to IV3 are AI group samples. NT1 to NT6 are SCNT group samples. (**B**) The volcanic map showing the distribution of DEGs. (**C**) GO enrichment analysis of DEGs between two groups. The red dots indicate the significantly up-regulated genes, and the blue dots indicate the significantly down-regulated genes in SCNT placentas; pathways related to lipid metabolism and function are marked by red frames.

The principal component analysis of the normalized RNA-Seq data showed that two groups of placentas are distributed in different regions (Figure 2A).

Compared with AI group, SCNT placentas have 2720 up-regulated and 1888 down-regulated genes. The top five up-regulated and the top five down-regulated genes were shown in the volcano map (Figure 2B). Interestingly, four of these ten DEGs with top fold change, including *TOMM22* [29], *ARHGEF6* [30,31], *GPC3* [32,33] and *BIRC07* [34,35] were reported to regulate apoptosis and growth.

GO enrichment analysis of the DEGs showed that many enriched pathways are related to lipid metabolism and function, including lipid metabolic process, high-density lipoprotein particle, protein–lipid complex, plasma lipoprotein particle, lipoprotein particle, lipid binding, phospholipid binding pathways (Figure 2C). This suggests that AI placentas and SCNT placentas are different in lipid metabolism and function.

**Figure 3:**
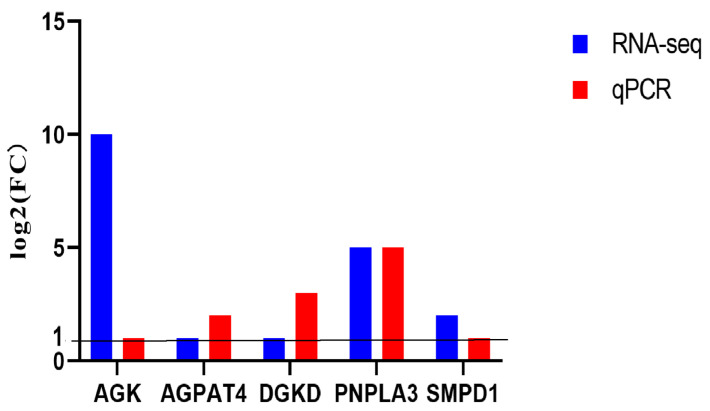
Verification of RNA-seq data by qPCR. Five genes involved in lipid metabolism and function were randomly selected for verification. FC: fold change in gene expression level (SCNT vs. AI).

To verify the RNA sequencing data, five genes related to lipid metabolism and function were randomly selected and their expression levels in two groups of placentas were measured by qPCR. The qPCR results matched with those detected by transcriptome sequencing (Figure 3), suggesting that the transcriptome data is reliable.

### 3.3. Lipidomics Analysis of AI and SCNT Fetal Placentas

**Figure 4:**
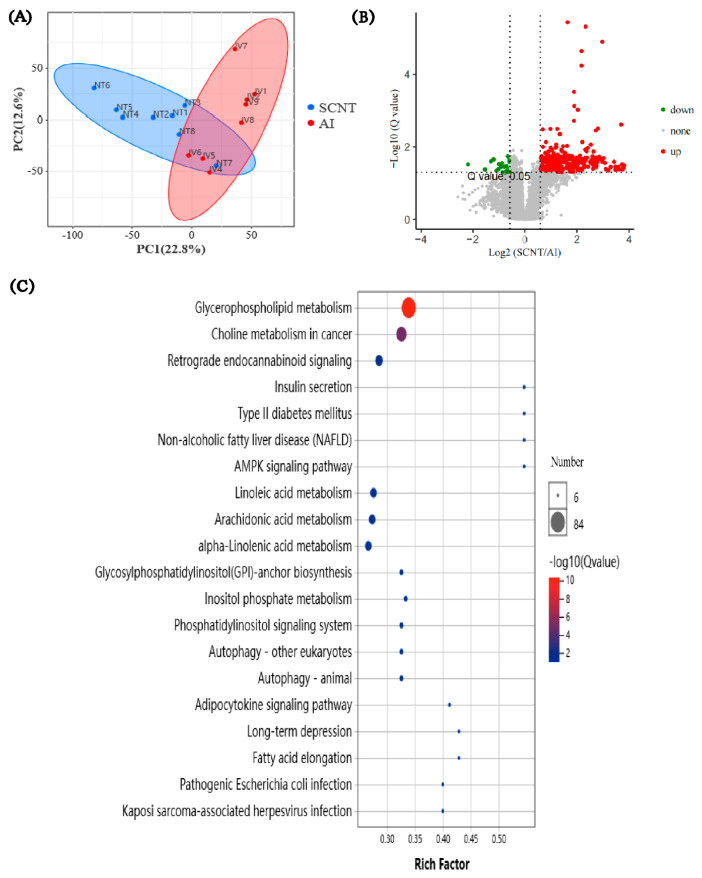
Lipidomics analysis of AI and SCNT fetal placentas. (**A**) PCA score scatter plot. IV1 to IV8 are AI group samples. NT1 to NT8 are SCNT group samples. (**B**) The volcanic map that shows the distribution of DEMs between two groups. (**C**) KEGG analysis of DEMs.

To further explore the key difference in metabolites between AI and SCNT placentas, these two groups of placentas were subjected to lipidomics analysis. The PCA score chart showed that two groups of placentas are distributed in different regions (Figure 4A). A total of 420 DEMs were found between SCNT and AI placentas. Compared with the AI group, 396 up-regulated DEMs and 24 down-regulated DEMs were found in SCNT placentas (Figure 4B).

**Figure 5:**
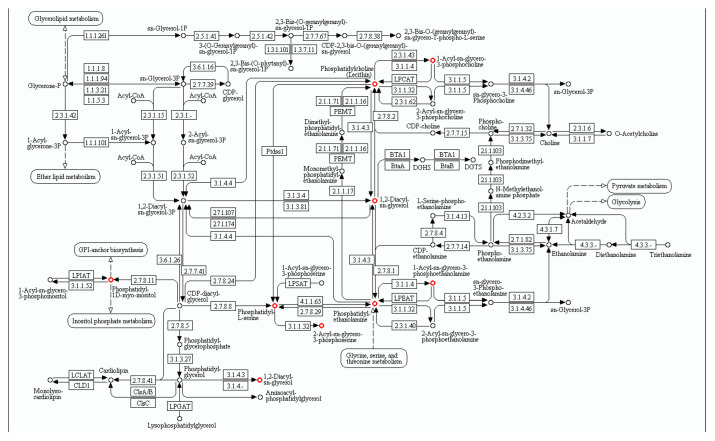
Metabolites in the glycerophospholipid metabolism pathway. The red dots indicate the significantly up-regulated metabolites in SCNT placenta.

KEGG enrichment analysis of the DEMs showed that the most enriched pathway is the glycerophospholipid metabolism pathway, and a total of 84 DEMs are involved in this pathway (Figure 4C). Surprisingly, the levels of all these 84 DEMSs were significantly increased in SCNT placentas, compared with the AI group (see Supplementary Table S2). Among the 84 DEMs up-regulated in SCNT placentas, 21 of them are different types of lysophosphatidylcholine (LPC) and 24 of them are different types of phosphatidylcholine (PC), which is the precursor of LPC in the glycerophospholipid metabolism pathway (Figure 5). These results indicate that the glycerophospholipid metabolism pathway is up-regulated, leading to an abnormally elevated level of LPC in SCNT placentas.

### 3.4. Effects of LPC on PTr2 Cell Proliferation and Migration

**Figure 6:**
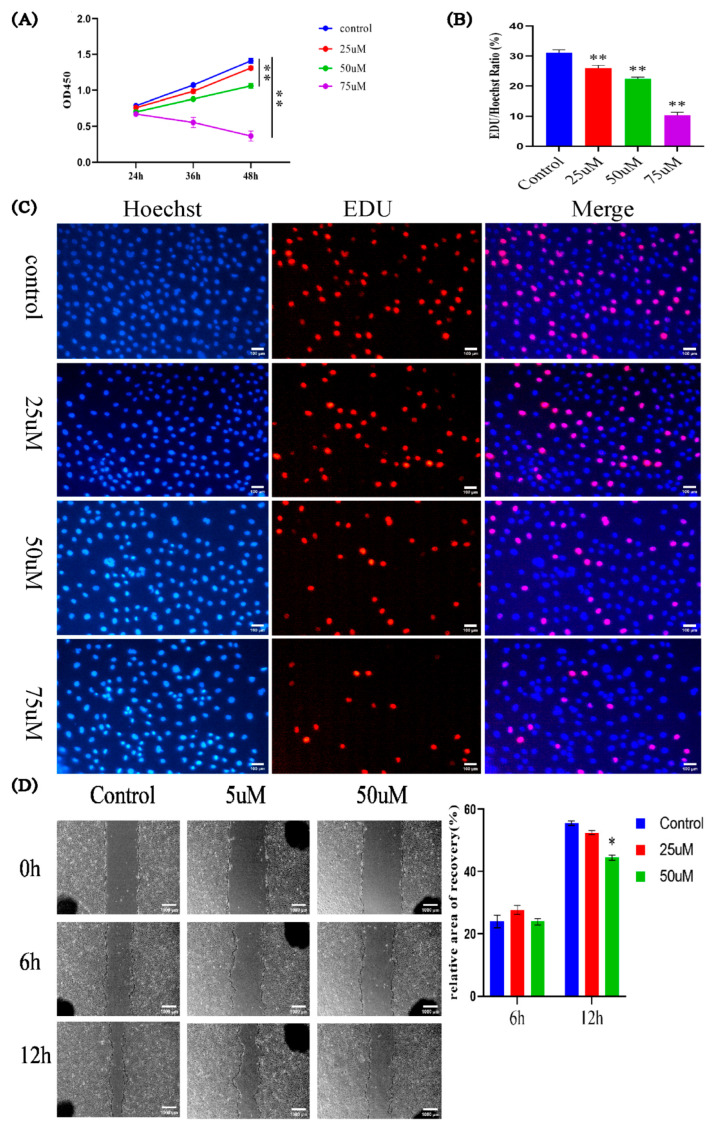
Effects of LPC on PTr2 cell proliferation and migration. CCK-8 (**A**) and EDU (**B**,**C**) measurement of proliferation of PTr2 cells treated with LPC. (**D**) Effects of LPC on PTr2 cell migration. Data are presented as mean ± SEM of three replicates. (* *p* < 0.05, ** *p* < 0.01).

LPC has been reported to be harmful for cell activity [36,37,38]. Our lipidomics analysis results showed that the LPC level in SCNT placentas was 22.7 times higher than that in AI placentas (Supplementary Table S2). Therefore, we decided to explore whether the elevated LPC level is related to the abnormal development of placenta in SCNT fetuses, by studying the effects of LPC on the proliferation and migration of a porcine trophoblast cell line called PTr2 [39]. PTr2 cells were treated with different concentrations of LPC (0 uM, 25 uM, 50 uM, 75 uM). The results of CCK-8 (Figure 6A) and EDU assays (Figure 6B,C) showed that LPC suppressed PTr2 cell proliferation in a concentration- and time-dependent manner. In addition, the results of the wound healing experiment showed that 12 h treatment with 50 uM of LPC significantly inhibited PTr2 cell migration (Figure 6D). These results suggest that LPC inhibits the proliferation and migration of PTr2 cells and the increased LPC level may be responsible for the impaired development of SCNT placentas.

## 4. Discussion

Our placental transcriptome data showed that the expression levels of two apoptosis-related genes, *TOMM22* and *ARHGEF6* were significantly up-regulated in SCNT placentas compared with AI placentas. Chae et al. [40] performed proteomic analysis of porcine placentas and showed that the protein abundances of Annexins and Hsp27, which are also related to apoptosis, were significantly up-regulated in SCNT placentas compared to the AI group. In addition, Ka et al. found a higher level of apoptosis in the placentas of 30- and 35-day-old SCNT fetuses than AI groups [41]. These findings imply that abnormal apoptosis of placentas may be a main cause of the developmental failure of SCNT embryos or fetuses. Our results showed that when treating with a high concentration of LPC (75 µM), the growth curve of porcine trophoblast cells goes down (see Figure 6A). This result suggests that a high level of LPC can induce apoptosis of porcine trophoblasts. In line with this, many studies have reported that LPC can induce apoptosis in various in vitro cultured cells [42,43,44,45,46]. In addition, our results indicated that the LPC level in pig SCNT placentas was 22.7 times higher than that in AI placentas. Therefore, the abnormally elevated LPC level in porcine SCNT placentas may induce trophoblast cell apoptosis.

Our KEGG analysis showed that a large number of DEGs between SCNT and AI placentas were enriched in lipid metabolism-related pathways. This indicates that the regulation of lipid metabolism is abnormal in SCNT placentas. The erroneous expression of lipid metabolism-related genes in SCNT placentas, as found by the transcriptome sequencing, may cause aberrant expression of metabolites involved in glyceroLphospholipid metabolism in SCNT placentas, which was found by the placental lipidomics analysis. The abnormal expression of lipid and/or glycerolphospholipid metabolites may lead to the abnormal growth of SCNT placentas, resulting in developmental defects of cloned fetuses.

We have demonstrated that the level of a critical metabolite in the glycerolphospholipid metabolism pathway, LPC, was 22.8 folds higher in SCNT placentas than in AI counterparts. LPC, also known as hemolysin, is a lipid biomolecule formed by the cleavage of phosphatidylcholine (PC) under the action of phospholipase A2 [47,48]. LPC has both pro-inflammatory and cytotoxic effects [36,37,38]. LPC acts through multiple receptors, such as G protein-coupled signal receptors [49], Toll-like receptors [50] and ion channels [51], to induce oxidative stress and inflammation [52], which changes the apoptosis and proliferation of cells [53,54,55]. Our results showed that LPC inhibits the proliferation and migration of PTr2 cells. Sun et al. found that LPC can induce apoptosis of H19-7 hippocampal progenitor cells by up-regulating Fasl expression [56]. Other studies also showed that LPC reduces the migration and proliferation of vascular endothelial cells and hinders vascular development by inhibiting Ras/ERK pathway [57,58]. A recent study has also shown that LPC inhibits cancer cell proliferation by increasing fatty acid oxidation and altering lipid metabolism [59].

Some anti-inflammatory drugs such as TSG, Web2170 and PD98059 can block the inflammatory response induced by LPC, thus protecting tissues and organs from LPC damage [60,61,62]. If the increased LPC abundance is responsible for the abnormal growth of pig SCNT placentas, then treating SCNT fetuses or their recipient mothers with LPC inhibitors may enhance the developmental ability of cloned pig fetuses. This idea could be tested in future studies.

## 5. Conclusions

In summary, elevated LPC levels in the placentas of cloned pig fetuses inhibit the proliferation and migration of trophoblast cells, resulting in impaired placental development, which leads to abnormal growth of cloned pig fetuses. This study uncovers a novel mechanism underlying the erroneous development of cloned pig fetuses. It will help to develop new methods to improve pig cloning efficiency.

## Data Availability

The sequence data in this study are openly available in the National Center for Biotechnology Information: https://dataview.ncbi.nlm.nih.gov/object/PRJNA1082775?reviewer=2kmq1vmo1kpr2p8r030qkaeu4e (accessed on 10 July 2025), accession number PRJNA1082775.

## Supplementary Materials

The following supporting information can be downloaded at https://www.mdpi.com/article/10.3390/jdb13040041/s1, Figure S1: Lipidomics quality control; Table S1: Primer of genes used in this article; Table S2: Comparison of the levels of 84 differentially expressed metabolites enriched in the glycerophospholipid metabolism pathway between SCNT and AI placentas..

## Abbreviations

The following abbreviations are used in this manuscript:

SCNT	Somatic cell nuclear transfer
AI	Artificial insemination
LPC	Lysophosphatidylcholine
PZM-3	Porcine fertilized egg medium 3
DEGs	Differentially expressed genes
GO	Gene Ontology
KEGG	Kyoto Encyclopedia of Genes and Genomes
QC	Quality control
TIC	Total ion chromatogram
OD	Optical density
PC	Phosphatidylcholine
