# Supplementary material for: Cloned Pig Fetuses Have a High Placental Lysophosphatidylcholine Level That Inhibits Trophoblast Cell Activity
Source: J Dev Biol. 2025 Nov 12;13(4):41. doi: 10.3390/jdb13040041 (PMC12641812; doi:10.3390/jdb13040041)
Supplement: Supplementary file 1 [file jdb-13-00041-s001.zip › jdb-3781082-supplementary.pdf]

**Table S1.** Primer of genes used in this article.

| Gene name | Base sequence(5'-3') | Base number（bp） | Product length（bp） |
|---|---|---|---|
| *AGPAT 4* | F：AACAAGCAGCTCTTCAGAAGGA | 22 | 197 |
| | R：CTCCAACCACAGAGAAAGTCGA | 22 | |
| *DGKH* | F：TCCGTCTTCAGGACTGGAAATG | 22 | 150 |
| | R：AGAAACCATCAGAATCGGTGCT | 22 | |
| *AGK* | F：ACTGGAGTTCTTCGAAGAGCAG | 22 | 174 |
| | R：CAGGGGAACTGTCTCTCCTTTC | 22 | |
| *PNPLA3* | F：CACGAACTTTCTTCACGTGGAC | 22 | 175 |
| | R：CCTGTCACAGATGCCATTCTCT | 22 | |
| *SMPD1* | F：GTGGATGAGTTCGAGGTGTTCT | 22 | 186 |
| | R：CAGGTTCAGGATGTAGGTCTCG | 22 | |

**Table S2.** Comparison of the levels of 84 differentially expressed metabolites enriched in the glycerophospholipid metabolism pathway between SCNT and AI placentas.

| MS2Metabolites | ratio （SCNT vs. AI） | t.test_p.value | VIP | regulated |
|---|---|---|---|---|
| LPC 16:0 | 2.22 | 0.026 | 2.78 | up |
| LPC 17:0 | 2.53 | 0.028 | 2.64 | up |
| LPC 18:2 | 1.88 | 0.026 | 3.44 | up |
| LPC 18:1 | 2.31 | 0.024 | 3.01 | up |
| LPC 18:0 | 2.86 | 0.022 | 2.78 | up |
| LPC 19:1 | 2.61 | 0.032 | 3.24 | up |
| LPC 17:0 | 2.53 | 0.042 | 2.40 | up |
| LPC 18:1 | 2.33 | 0.034 | 2.82 | up |
| LPC 18:0 | 2.85 | 0.023 | 2.84 | up |
| LPC O-18:1 | 2.58 | 0.007 | 3.00 | up |
| LPC 14:0-SN1 | 1.74 | 0.037 | 2.34 | up |
| LPC 15:0-SN1 | 1.99 | 0.036 | 2.58 | up |
| LPC 16:1-SN1 | 1.85 | 0.041 | 2.49 | up |
| LPC 16:0-SN1 | 2.24 | 0.036 | 2.80 | up |
| LPC 17:1-SN1 | 2.07 | 0.026 | 2.91 | up |
| LPC 17:0-SN1 | 2.53 | 0.028 | 2.66 | up |
| LPC 18:1-SN1 | 2.32 | 0.020 | 3.05 | up |

| | | | | |
|---|---|---|---|---|
| LPC 18:0-SN1 | 2.86 | 0.026 | 2.79 | up |
| LPC 19:1-SN1 | 2.60 | 0.027 | 3.05 | up |
| LPC 20:2-SN1 | 2.45 | 0.025 | 2.84 | up |
| LPC 20:1-SN1 | 2.91 | 0.020 | 3.15 | up |
| PC O-18:1_2:0 | 2.92 | 0.022 | 2.92 | up |
| PC 18:2_18:2 | 5.40 | 0.003 | 2.06 | up |
| PC 18:1_18:2 | 5.75 | 0.017 | 1.79 | up |
| PC 18:0_18:1 | 6.37 | 0.029 | 1.72 | up |
| PC 16:1_18:1 | 5.72 | 0.038 | 1.51 | up |
| PC 17:1_18:2 | 5.56 | 0.022 | 1.77 | up |
| PC 18:2_18:2 | 5.42 | 0.029 | 2.48 | up |
| PC 18:1_18:2 | 5.77 | 0.017 | 2.16 | up |
| PC 18:0_18:1 | 6.37 | 0.044 | 1.71 | up |
| PC 18:0_19:1 | 6.51 | 0.035 | 1.97 | up |
| PC 18:1_20:3 | 5.84 | 0.046 | 2.18 | up |
| PC 18:1_20:2 | 6.09 | 0.030 | 1.77 | up |
| PC 18:1_22:6 | 5.54 | 0.025 | 1.74 | up |
| PC 21:1_21:1 | 6.94 | 0.041 | 1.80 | up |
| PC 11:0_32:2 | 7.07 | 0.035 | 1.75 | up |
| PC 22:3_22:3 | 6.28 | 0.042 | 1.92 | up |
| PC 22:1_22:1 | 7.19 | 0.044 | 1.86 | up |
| PC 31:0 | 5.85 | 0.020 | 1.94 | up |
| PC 37:1 | 6.52 | 0.041 | 1.76 | up |
| PC 36:1 | 6.36 | 0.042 | 1.92 | up |
| PC 35:3 | 5.56 | 0.045 | 1.68 | up |
| PC 38:3 | 6.10 | 0.033 | 1.88 | up |
| PC 39:2 | 6.50 | 0.045 | 1.40 | up |
| PC 40:5 | 6.00 | 0.046 | 1.35 | up |
| LPE O-17:1 | 2.90 | 0.041 | 2.05 | up |
| LPE O-18:2 | 2.65 | 0.017 | 2.73 | up |
| LPE O-18:1 | 3.28 | 0.026 | 2.57 | up |
| LPE O-17:1 | 2.90 | 0.029 | 2.18 | up |
| LPE O-18:2 | 2.64 | 0.020 | 2.81 | up |
| LPE O-18:1 | 3.27 | 0.041 | 2.47 | up |
| LPE 16:0 | 2.32 | 0.025 | 2.68 | up |
| LPE 17:0 | 2.64 | 0.024 | 2.51 | up |
| LPE 18:1 | 2.40 | 0.022 | 3.27 | up |
| LPE 18:0 | 2.99 | 0.013 | 2.94 | up |
| LPE 19:1 | 2.71 | 0.025 | 3.21 | up |
| LPE 20:1 | 3.04 | 0.028 | 3.55 | up |
| LPE 18:1 | 2.41 | 0.020 | 3.22 | up |
| LPE 19:1 | 2.70 | 0.025 | 3.07 | up |
| LPS 18:0 | 2.50 | 0.003 | 3.39 | up |
| PS 16:0_18:1 | 5.71 | 0.036 | 1.56 | up |

| | | | | |
|---|---|---|---|---|
| PS 18:1_18:1 | 5.74 | 0.012 | 2.00 | up |
| PI 18:1_18:1 | 5.68 | 0.022 | 2.50 | up |
| PI 18:1_20:4 | 5.26 | 0.029 | 1.56 | up |
| DG 16:0_18:1 | 6.73 | 0.043 | 1.51 | up |
| DG 17:1_18:1 | 6.59 | 0.048 | 1.81 | up |
| DG 18:1_18:1 | 6.75 | 0.046 | 2.03 | up |
| DG 18:1_19:1 | 6.89 | 0.042 | 2.15 | up |
| PE O-17:1_18:1 | 6.51 | 0.044 | 1.82 | up |
| PE O-18:2_18:1 | 6.35 | 0.041 | 1.74 | up |
| PE O-20:1_18:1 | 6.96 | 0.017 | 2.16 | up |
| PE O-17:1_18:1 | 6.49 | 0.023 | 1.72 | up |
| PE O-13:0_5:0 | 2.98 | 0.011 | 2.98 | up |
| PE O-18:2_18:1 | 6.34 | 0.042 | 1.85 | up |
| PE 17:1_17:1 | 5.80 | 0.036 | 1.97 | up |
| PE 18:0_18:1 | 6.47 | 0.049 | 1.93 | up |
| PE 36:3 | 5.84 | 0.041 | 2.50 | up |
| PI 34:2 | 5.28 | 0.018 | 2.04 | up |
| PI 37:2 | 5.82 | 0.043 | 2.11 | up |
| PI 36:1 | 6.01 | 0.035 | 1.94 | up |
| PI 36:1 | 6.01 | 0.048 | 2.10 | up |
| PI 38:5 | 5.27 | 0.018 | 1.62 | up |
| PI 38:3 | 5.82 | 0.046 | 1.59 | up |
| PS 18:1_18:1 | 5.79 | 0.018 | 1.98 | up |

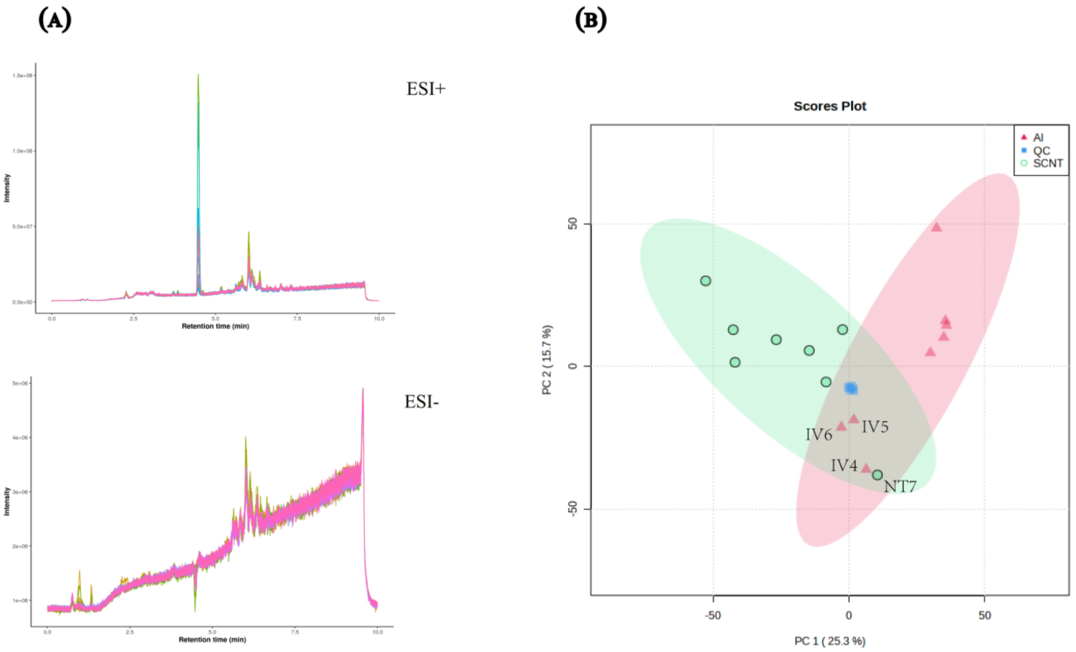


**Figure S1.** Lipidomics quality control. (A) Total Ion Chromatogram(TIC). (B) PCA score scatter plot.
